# Heat Transfer Enhancement of Controllable Aspect Ratio Fractal Channel

**DOI:** 10.3390/mi14091693

**Published:** 2023-08-29

**Authors:** Zhichao Men, Wenjiong Chen

**Affiliations:** State Key Laboratory of Structural Analysis, Optimization and CAE Software for Industrial Equipment, Dalian University of Technology, Dalian 116024, China; mzc_0913@163.com

**Keywords:** heat sink, fractal channel, controllable aspect ratio (AR), parameterized modeling method, performance

## Abstract

Thermal management technology is a major challenge in high-end equipment. The demand for high-efficiency heat sinks has increased. In this study, a controllable aspect ratio (AR) fractal channel (CARFC) heat sink is proposed to enhance thermal performance. First, a parameterized modeling method for the CARFC is constructed. Fractal networks are constructed using control points and bifurcation points. The geometric size of each level channel is determined by considering the AR of each level channel. A mathematical relationship is established between the two parts. Under constant heat flow boundary, the effect of aspect ratio on the fractal channel performance is studied by numerical simulation. The influence of the inlet AR on the performance of the fractal channels is studied. Then, the impact of the AR of each level channel on the performance of the CARFC is studied. The results show that the AR of the inlet has an obvious effect on the performance of the fractal channel. The CARFC results show that the AR of each level channel influences the thermal performance of the heat sink, especially the aspect ratio $k_{0}$ and $k_{1}$. Compared with only changing the aspect ratio of the inlet, the CARFC has better performance; the peak temperature and temperature difference are reduced by 9.62% and 26.57%, respectively. The CARFC requires less coolant to meet the same thermal demand, which is of great significance in the development of lightweight equipment.

## 1. Introduction

High-end equipment generates a large heat flow during operation. It is harmful to structural safety and operational performance. The heat-dissipation performance of modern high-power compact equipment directly affects its service life [1,2]. Thus, thermal management technologies have become increasingly important.

Microchannel heat sinks are widely used in high-end equipment thermal management owing to their large channel heat transfer area, high heat transfer efficiency, and lightweight. At the end of the 20th century, Tuckerman and Pease designed the electronic microchannel heat sink [3]. With the development of microchannel heat exchangers, the influence of microchannel geometry on performance is studied, including the channel cross-sectional shape [4], aspect ratio [5,6,7], channel distance [8], channel height [9], and channel width [10].

The aspect ratio (AR) of the radiator has an important influence on the flow and heat transfer performances. Peng and Peterson conducted an experimental analysis of a microchannel [11]. It was found that the AR greatly affects the hydraulic and thermal properties of the microchannels. Kewalramani studied a trapezoidal microchannel and found that the Nusselt number increased as the AR increased [12]. Kim studied rectangular microchannels experimentally; it was found that when the AR decreased from 1.0 to 0.25, the Re increased [13]. Sahar performed numerical simulations of rectangular microchannels and found that the thermal performance enhanced when the channel width increased at a constant flow rate [5]. Soheil found that the Nusselt number and pressure drop increased with a decrease in the channel height [9]. Thus, studies on channel ARs are essential.

A fractal channel is an innovative channel configuration inspired by nature. The performance and structural parameters of fractal channels have been studied by several researchers. The fractal channel exhibited a better performance. Compared with straight array channels, fractal channels have the advantages of a higher heat exchange rate [14], smaller maximum wall temperature [15], more uniform temperature distribution [16], higher thermal efficiency [17], lower pressure drop [14,15], and higher comprehensive performance [18]. Compared with the spiral channel, the fractal channel heat sink has a smaller maximum wall temperature and total pressure drop [19]. The parameters of the fractal channel have been studied, including the number of layers, arrangements, cavities and ribs, bifurcation angle, hydraulic diameter ratio, and bifurcation levels. Compared with single-layer fractal channels, multilayer fractal channels provide better heat transfer performance, including a smaller peak temperature [20], smaller temperature difference [20], smaller thermal resistance [21], and smaller irreversible loss [22]. The multilayer Y-shaped fractal channel exhibited better flow performance [20,21]. Yan [23] studied a fractal truncated double-layer heat sink. It was found that the temperature uniformity increased by 24–30%, and the pump power was reduced. Liu [24] proposed three different Y-shaped fractal channel arrangements that effectively improved the heat dissipation performance. In addition, ribs and cavities [25], small bifurcation angles [26], variable hydraulic diameters, and increased bifurcation levels [27] can enhance the heat transfer performance of fractal channels. The aspect ratio (AR) has an obvious influence on the performance of the fractal channel. Yu [28] investigated the effects of three ARs (1, 0.5, and 0.333) on the thermal and hydraulic performance of fractal channels by changing the inlet width of the fractal channel. It was found that when the AR was 0.333, the channel had the best flow performance and the smallest average heat transfer coefficient.

In previous studies, it was found that the aspect ratio (AR) is a significant structural parameter in the research of fractal channels, and has an obvious influence on the performance of the channel. However, for the study of the aspect ratio of the fractal channel, AR is controlled by changing the channel width and is limited to the inlet. Therefore, a parameterized modeling method of controllable AR fractal channel (CARFC) is constructed in this paper. Keeping the section area constant, the aspect ratio can be controlled by changing the height and width of the channel simultaneously, and the aspect ratio of each level of the fractal flow channel can be controlled. At the constant heat flux boundary, the influence of the inlet AR on the heat transfer performance of the fractal channel is studied. Then, the influence of the aspect ratio of each level of the fractal channel on the heat transfer performance is also studied.

## 2. Parameterized Modeling Method of Fractal Channel with Controllable Aspect Ratio

**Figure 1:**
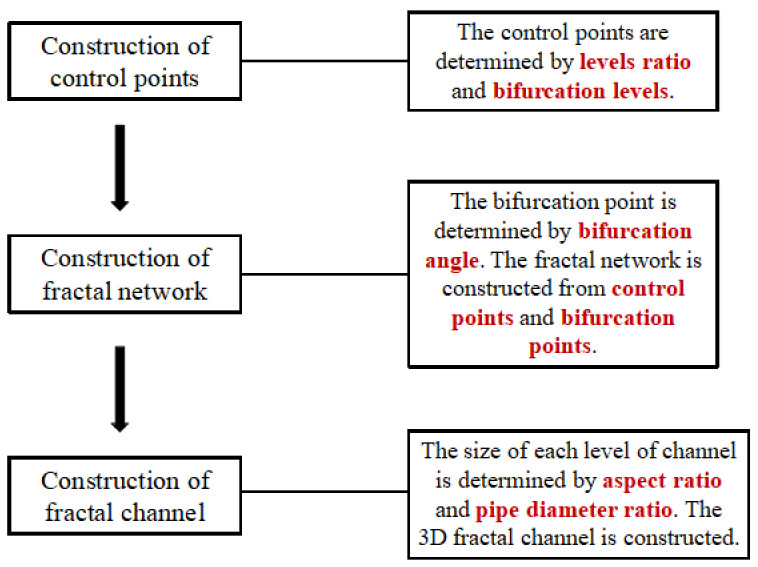
FCPM method.

In our previous study [29], a fractal channel parameterized modeling method (FCPM method) is proposed. However, the previous study has some shortcomings: the description of the aspect ratio is limited to the inlet cross-section, and the aspect ratio of other channels could not be precisely controlled. To date, studies on the impact of AR on the thermal performance of fractal channels have been limited to the inlet cross-section. This section presents an improved FCPM method with a controllable aspect ratio. The FCPM method is shown in Figure 1. The method consists of three steps. The first step is the construction of control points. The second step is the construction of a fractal network. The third step is the construction of fractal channels. The three steps are described in detail in Section 2.1, Section 2.2 and Section 2.3.

### 2.1. Construction of Control Points

The fractal channel is composed of several Y-shaped structures. A Y-shaped structure can be determined according to three control points at the end and a middle bifurcation point. Therefore, we construct control points in this section.

**Figure 2:**
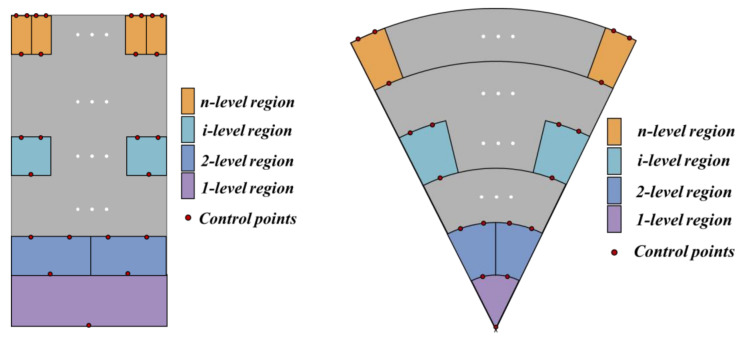
Control points.

Control points are constructed to determine the location and number of inlets and outlets at each level fractal channel. A common heat sink shape can be composed of a rectangle and a sector element. Control points are determined for the rectangular and sector plates, as shown in Figure 2. The length of each level of the fractal channel is determined by the level ratio, as shown in Equation (1). The number of inlets and outlets at each level of the fractal channel is determined according to the bifurcation levels, which are $2^{n-1}$ and $2^{n}$, respectively. In this section, the end control points are constructed, as shown in the red points in Figure 2.

(1) $$\left\{\begin{array}{l} l=l_{1}+l_{2}+\cdots +l_{i}+\cdots +l_{n}\\ l_{i+1}/l_{i}=\beta \end{array}\right.$$

where β is the levels ratio, which is the ratio of the length of the next level to the length of the last level.

### 2.2. Construction of Fractal Network

The construction of end control points is completed in Section 2.1. Therefore, the fractal network can be completed only by completing the construction of middle bifurcation points.

**Figure 3:**
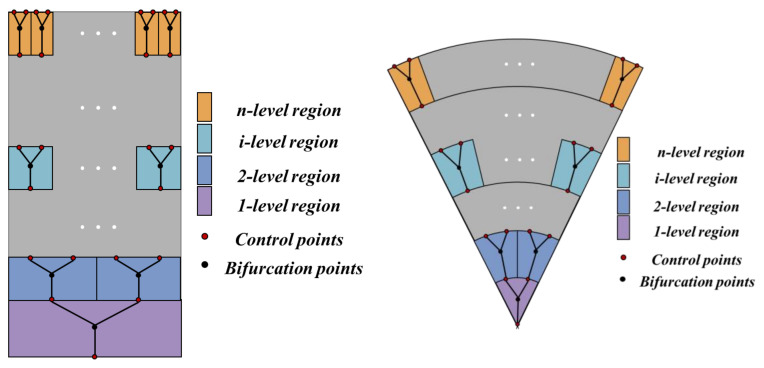
Bifurcation points.

The purpose of constructing a network is to determine the contours of the fractal channel. First, the bifurcation point is determined using Equation (2) according to the bifurcation angle, as shown in Figure 3 black points. Then, the bifurcation points and control points are connected to the complete construction of the fractal network, as shown in Figure 3.

(2) $$\left\{\begin{array}{l} x_{ij}=\frac{W}{2^{n}}+N\cdot \frac{W}{2^{n-1}},\;N=0,1,\cdots ,2^{n-1}-1\\ y_{ij}=\sum_{i=0}^{n}l_{i}-\frac{w_{i}}{4\cdot \tan\left(\alpha /2\right)} \end{array}\right.$$

where W is the substrate width, and α is the bifurcation angle.

### 2.3. Construction of Fractal Channel

**Figure 4:**
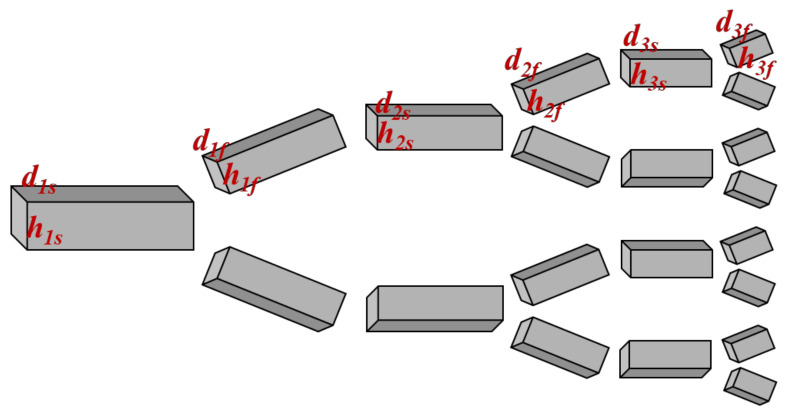
Fractal channel model.

The fractal network is constructed in Section 2.2. Then, the construction of the channel along the fractal network can complete the establishment of the fractal channel. Therefore, the width and height of the channel need to be determined in this part. The width and depth of each level of the rectangular cross-section fractal channel are determined using Equations (3) and (4). Finally, according to the fractal network and flow channel size, the three-dimensional fractal channel was constructed, as shown in Figure 4. This part is the focus of this work. In the previous work [29], the aspect ratio is only limited to the aspect ratio (k) of the entrance.

(3) $$d_{inlet}=\sqrt{\frac{V}{l_{1s}/k_{0}+2\lambda^{2}\left(l_{1f}+l_{2s}\right)/k_{1}+\cdots +2^{i-1}\lambda^{2\left(i-1\right)}\left(l_{\left(i-1\right)f}+l_{is}\right)/k_{i-1}+\cdots +2^{n}\lambda^{2n}l_{nf}/k_{n}}}$$

(4) $$\left\{\begin{array}{l} d_{is}=d_{1s}\lambda^{i-1}\\ d_{if}/d_{is}=\lambda \\ h_{i}=d_{i}/k_{i-1}\\ d_{i}=d_{is}\\ h_{i}=h_{is} \end{array}\right.$$

where λ is the Pipe diameter ratio, which means the ratio of the width of the next level channel to the width of the last level channel.

## 3. Simulation Models

### 3.1. Physical Model

Using the parameterized fractal channel modeling method proposed in our previous study and an improved parameterized modeling method, two types of fractal channels are constructed. The effects of the inlet aspect ratio and all levels of the channel aspect ratio on fractal channel performance are discussed, respectively.

**Figure 5:**
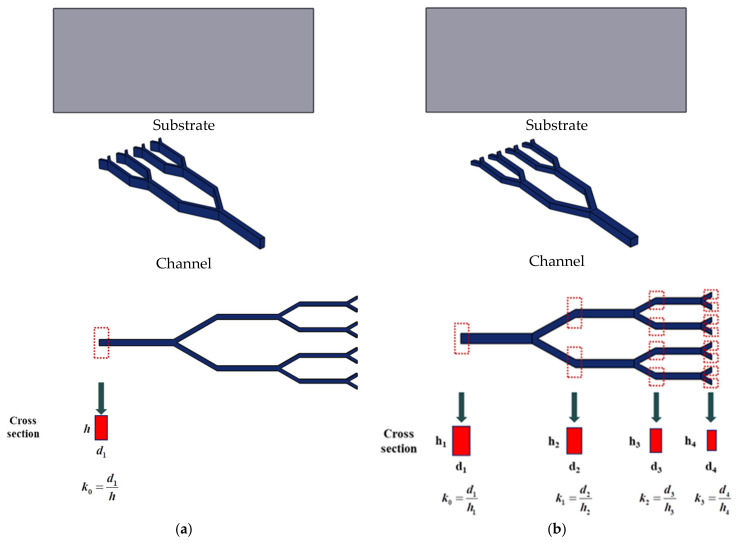
Geometric models of the fractal channel: (**a**) IARFC. (**b**) CARFC.

The aspect ratio (AR) models of the two types of fractal channels are shown in Figure 5. In model (a) (IARFC), the fractal channel is constructed by the previous FCPM method; only the AR at the inlet section is controlled. In model (b) (CARFC), the fractal channel is constructed using an improved parameterized modeling method; the AR of all levels channel can be controlled. Y-shaped fractal channels with the AR ranging from 0.5 to 2.9 are designed. The geometric information is presented in Table 1. The substrate materials are presented in Table 2. The substrate material is aluminum. Water is used as the heat-exchange medium.

### 3.2. Numerical Simulation Model

In order to study the controllable aspect ratio (AR) fractal channel, COMSOL Multiphysics is used for the numerical simulation. To simplify the control equation, the following hypotheses are made:

(1) The flow is incompressible.
(2) The flow is laminar.
(3) The flow is steady.
(4) The effect of gravity is negligible.
(5) The viscous dissipation is ignored.
(6) Negligible radiation effects.

The control equations:

Continuity equation:

(5) ρ∇⋅u=0

Momentum equation:

(6) $$\rho \left(u\cdot \nabla \right)u=\nabla \cdot \left[-pΙ+Κ\right]+F$$

Energy equation:

(7) $$\rho C_{p}u\cdot \nabla T+\nabla \cdot q=Q$$

(8) q=−k∇T

To solve the control equations, COMSOL Multiphysics is used. COMSOL uses the finite element method to solve basic partial differential equations, discretizing the modeling domain into smaller and simpler domains, which are called units. The equations (shape functions) in each cell can have different orders. Each physical field interface has its own unique discretization setting that controls the order of the form function used on the dependent variable. In general, the problems of fluid flow and transfer are usually first-order linear discretization. The temperature field is solved by linear elements, the flow field is solved by first-order discretizations (P1 + P1) using linear interpolation. In the solving process, a segregated approach and a direct solver (PARDISO) are used. The conservation equations are regarded as converging when the residuals are less than 10^−6^.

### 3.3. Boundary Conditions

Table 3 shows the boundary conditions for the following studies. All fractal channel heat sink walls are the fluid–solid conjugated heat-transfer walls. The internal interface walls are defined as non-slip boundary conditions. The bottom wall of the heat sink is set with a uniform heat flux value of 500,000 W/m^2^. The upper wall and sidewall of the heat sink have no heat inflow, that is, the adiabatic boundary. The inlet temperature of the water is 293.15 K. The mass flow rates of the fractal channel inlet are 0.5, 1.0, and 1.5 g/s. The pressure of the outlet is 0 Pa.

### 3.4. Grid Dependence

**Figure 6:**
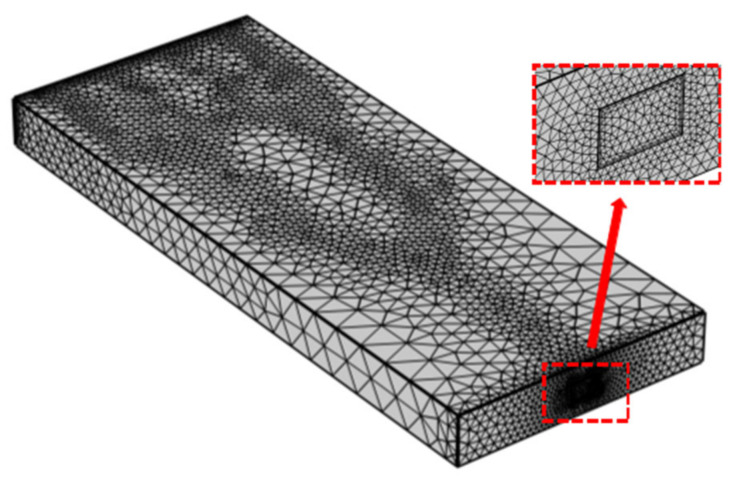
Mesh 2.

Unstructured grids are used for numerical simulation in this paper. A tetrahedral mesh is selected. The reason for this is that this type of mesh has good geometric adaptability. To reduce the influence of the grid on the numerical simulation results, three kinds of grids with different numbers and sizes are used to study the dependence of the numerical results on grids. Mesh 1 has 366,424 grids and a minimum size of 0.0693 mm. Mesh 2 has 1,120,504 grids and a minimum size of 0.0346 mm. Mesh 3 has 4,528,940 grids and a minimum grid size of 0.0139 mm. The peak temperatures of the three mesh models are compared under different mass flow rates. Compared to Mesh 1, the average error of Mesh 2 is 3.277%. Compared to Mesh 2, the average error of Mesh 3 is 0.6067%. The peak temperature changes slowly with the increase in the mesh element number and the decrease in mesh size; thus, the effect of the grids can be ignored. Thus, the amount of calculation can be reduced and the accuracy of the results can be ensured using Mesh 2. The grid is illustrated in Figure 6.

### 3.5. Performance Index

In the study of the aspect ratio (AR), many performance indices are used, including the Nusselt number (Nu), thermal resistance ($R_{t}$), temperature difference (ΔT), entropy generation rate ($S_{ht}$), pressure drop (ΔP), and figure of merit (FOM).

(1) Nusselt number (Nu) [30]:
  (9) $$Nu=\frac{q\cdot D_{h}}{k_{l}\cdot \left(T_{ave}-\frac{T_{in}+T_{out}}{2}\right)}$$
  where q is the heat flux of the bottom; $D_{h}$ is the hydraulic diameter; $k_{l}$ is the fluid thermal conductivity; $T_{ave}$ is the heat sink average temperature; $T_{out}$ is the outlet temperature; and $T_{in}$ is the fluid temperature at the inlet.
(2) Thermal resistance ($R_{t}$) [22]:
  (10) $$R_{t}=\frac{T_{\max}-T_{0}}{A_{h}\cdot q}$$
  where $T_{\max}$ is the peak temperature of the mico-channel heat sinks; $T_{0}$ is the inlet temperature $T_{in}$, and $A_{h}$ is the bottom area of the heat sink.
(3) Entropy generation rate ($S_{ht}$) [22]:
  (11) $$S_{ht}=\frac{qA_{h}\left(T_{h}-T_{m}\right)}{T_{h}T_{m}}$$
  where $T_{h}$ is the heated surface average temperature, and $T_{m}$ is the fluid average temperature.
(4) Temperature difference (ΔT):
  (12) $$\Delta T=T_{\max}-T_{min}$$
  where $T_{\min}$ is the minimum temperature of heat sinks
(5) Pressure drop (ΔP):
  (13) $$\Delta P=P_{in}-P_{out}$$
(6) Figure of merit (FOM) [31,32]
  (14) $$\begin{array}{l} FOM=\frac{h_{1}/h_{2}}{{\left(W_{pump1}/W_{pump2}\right)}^{1/3}}\\ W_{pump}=\Delta P\times V_{\mathrm{in}} \end{array}$$

The comprehensive performance is indicated by the FOM. Where h is the average coefficient of heat transfer, $W_{pump}$ is the pumping power, and $V_{\mathrm{in}}$ is the volume flow rate.

### 3.6. Verification of Numerical Model

**Figure 7:**
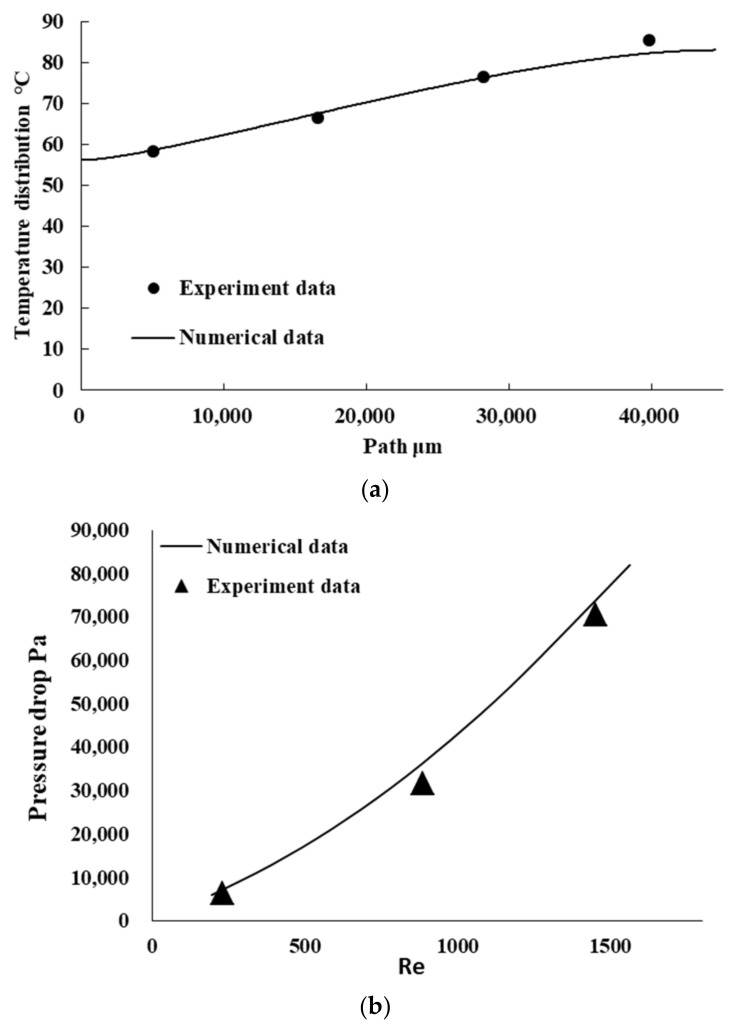
Verification of numerical model: (**a**) Temperature distribution of numerical and experimental results. (**b**) Temperature distribution of numerical and experimental results.

To verify the effectiveness of the simulation model, we compare it with an experiment [33]. Based on the experiments in the literature, we construct a numerical model. The heat sink is made of copper and the coolant is water. The heat sink channel is composed of a series of microchannels, and the inlet size is 231 μm × 713 μm. At Re of 226 and heat flux of 100 $W/{\mathrm{cm}}^{2}$, the temperature distribution of the experiment and numerical simulation is compared at the position of 3175 μm from the bottom of the radiator. At the heat flux of 100 $W/{\mathrm{cm}}^{2}$, the pressure drop of the experimental and numerical simulation with different Re is compared. Figure 7a shows a comparison of the temperature distributions, including numerical simulation and experiment results. Figure 7b shows a comparison of the pressure drops between numerical simulation and experiment. The numerical simulation results are in good agreement with the experimental results. Thus, the reasonableness and validity of the numerical results are verified.

## 4. Results and Discussion

### 4.1. Effect of Inlet Aspect Ratio on Performance of Fractal Channel

**Figure 8:**
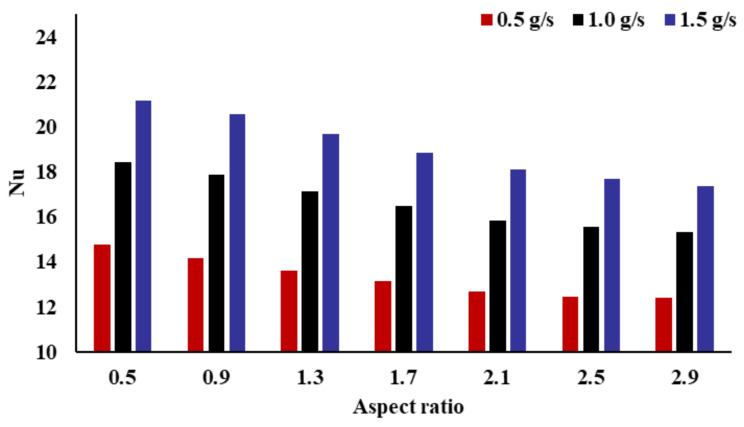
Effect of the inlet AR on Nusselt number.

Figure 8 illustrates the connection between the Nusselt number (Nu) and the AR. The total heat transfer performance is described by the Nusselt number. It can be seen from the resulting figure that the AR has an evident impact on the Nusselt number. With the increase in the AR, the Nusselt number is decreased monotonically, which means that the total heat-transfer performance of the fractal channel radiator decreases. From the definition of the Nusselt number in Formula (9), it can be seen that the Nusselt number has an important relationship with the average temperature of the heat sink and hydraulic diameter. With the increase in AR, the hydraulic diameter shows a decreasing trend, the average temperature of the heat sink increases first and then decreases, and the change is very small in the decreasing stage, which leads to a decrease in the Nusselt number. With different mass flow rates, the total impact trend of the AR on the Nusselt number is consistent. When the AR is 0.5, the Nusselt number of the radiator is the largest. When the AR is 2.9, the Nusselt number of the radiator is the smallest. When the mass flow rate is 0.5, 1.0, and 1.5 g/s, the Nusselt number of the fractal channel with an AR of 0.5 increased by 19.38%, 20.18%, and 21.77%, respectively, compared with the fractal channel with an AR of 2.9. As the mass flow rate increases, the impact degree of the AR on the Nusselt number increases.

**Figure 9:**
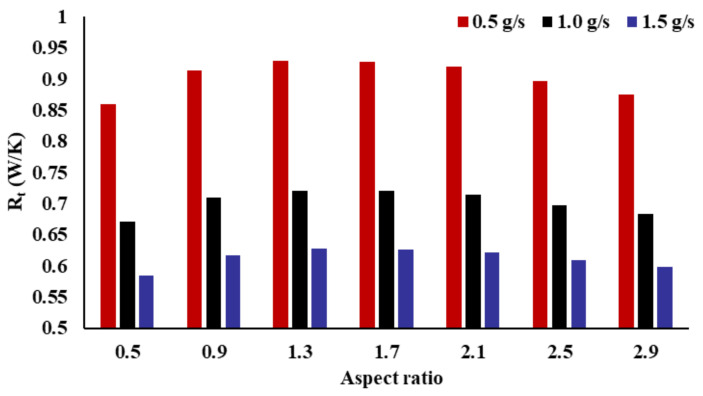
Effect of the inlet AR on thermal resistance.

Figure 9 shows the connection between the thermal resistance ($R_{t}$) and the AR. As shown in this picture, the AR has an evident impact on the thermal resistance. The thermal resistance exhibits a non-monotonic tendency of first increasing and then decreasing, with an increase in the AR. When the AR is 0.5, the thermal resistance is minimal. When the AR is 1.3, the thermal resistance is at its maximum. With different mass flow rates, the differences in thermal resistance of the two AR radiators are 0.06908, 0.04963, and 0.04210, respectively. When the AR is 0.5, the thermal resistance of the heat sink decreases by 7.44%, 6.88%, and 6.71%, respectively, compared with an AR of 1.3. When the mass flow rate is lower, the AR has a greater impact on thermal resistance. The AR has less influence on thermal resistance as the mass flow rate at the inlet increases from 0.5 to 1.5 g/s.

The thermal resistance ($R_{t}$) and Nusselt number ($N_{u}$) are important indices for appraising the heat-transfer performance of radiators. The ability of a radiator to control the peak temperature is described by thermal resistance. The Nusselt number describes the total thermal performance of the radiator. In Figure 8 and Figure 9, the AR has an obvious influence on the thermal performance. However, the impact of the AR on the Nusselt number and thermal resistance is inconsistent. The relationship between the Nusselt number and the AR is monotonous, the Nusselt number decreases as the AR increases. With an increase in the AR, the thermal resistance exhibits a non-monotonic trend of first increasing and then decreasing.

**Figure 10:**
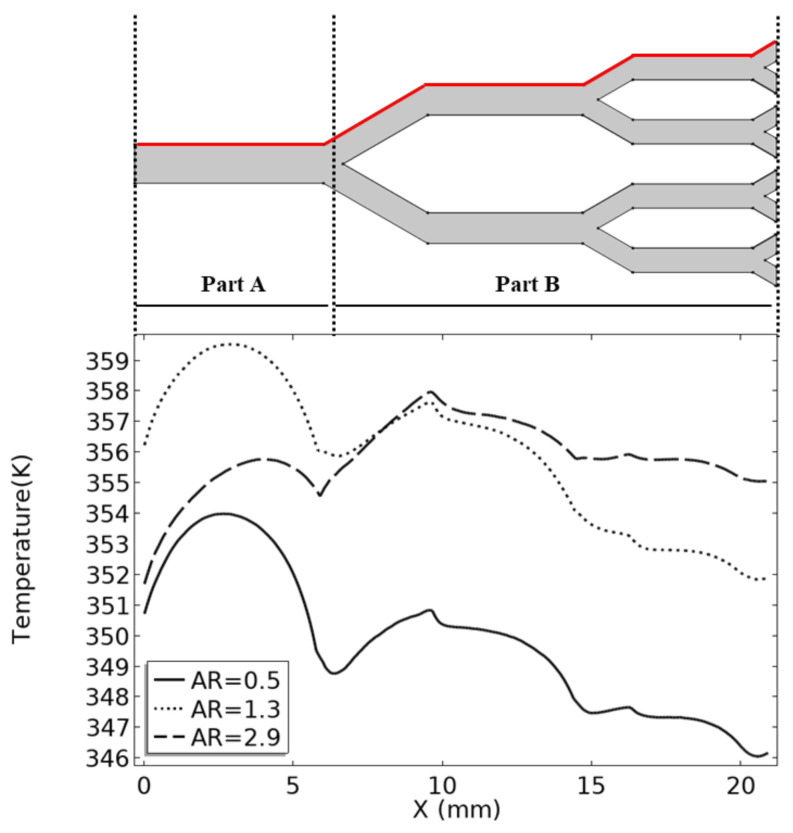
Fluid boundary temperature distribution.

In order to analyze this phenomenon, the fluid boundary temperature distribution of fractal channels with aspect ratios of 0.5, 1.3, and 2.9 is shown in Figure 10. The channel with an AR of 0.5 has the lowest temperature at the fluid boundary in the entire channel. Thus, the thermal performance of the radiator is the best in this case. In part A of the channel, the fluid boundary temperature with an AR of 2.9 is only greater than that of the channel with an AR of 0.5. Thus, in part A, the heat transfer performance of the channel with an AR of 2.9 is second only to that of the fractal channel with an AR of 0.5. In part B, the boundary temperature in the fractal channel with an AR of 2.9 rises sharply; eventually, the boundary temperature becomes the highest in all cases. Thus, when the fractal channel with an AR of 2.9 is in Part A, the thermal performance is better; in Part B, the thermal performance is reduced. In the rectangular plate, part A of the fractal channel had the greatest influence on the high-temperature region. Thus, the thermal resistance of the radiator with a channel AR of 2.9 is only higher than that of the radiator with a channel AR of 0.5. With the increase in the AR, the thermal performance of Part A first decreased and then increased, and the thermal performance of Part B decreased monotonously. Thus, with the increase in the AR, the two evaluation indices of thermal performance showed different trends.

**Figure 11:**
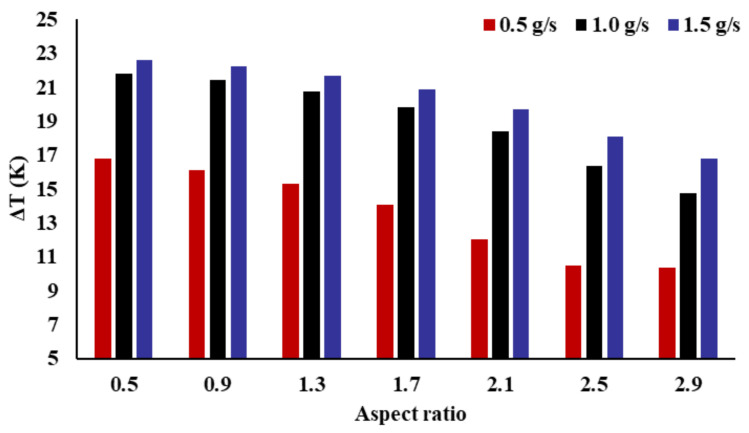
Effect of the inlet AR on temperature difference.

Figure 11 shows the connection between the temperature difference (ΔT) and AR of the radiator channel. It is observed that the AR has an evident impact on the radiator temperature difference. It was found that the temperature difference decreased with the increase in the AR. With different mass flow rates, the maximum differences are 6.43 K, 7.04 K, and 5.84 K, respectively. Compared with the channel radiator with an AR of 0.5, the temperature difference of the channel radiator with an AR of 2.9 is reduced by 38.31%, 32.27%, and 25.81%, respectively. As the mass flow rate increases, the reduction in the heat sink temperature difference decreases. With the increase in mass flow rate, the impact of the AR on temperature difference decreases.

**Figure 12:**
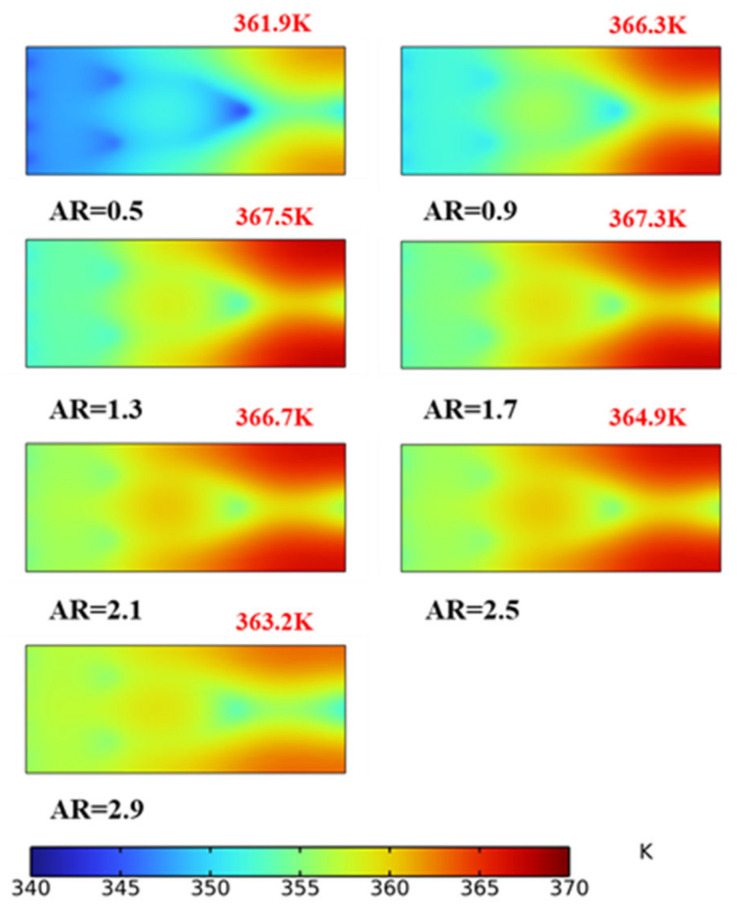
Temperature distribution.

**Figure 13:**
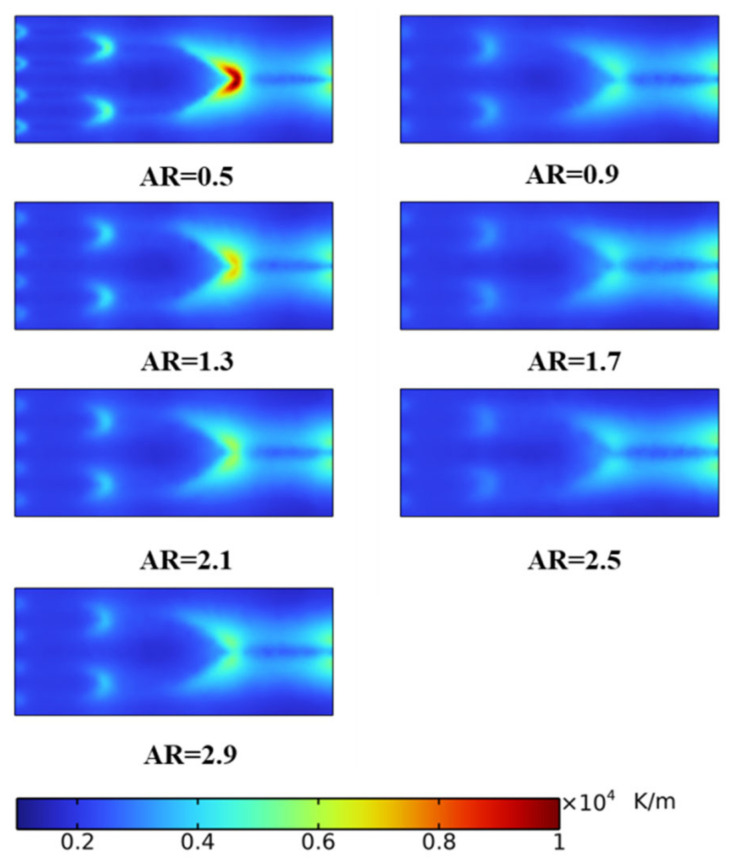
Temperature gradient distribution.

To better understand the influence of the AR on the fractal channel heat sink, the temperature field and temperature gradient on the radiator surface are shown in Figure 12 and Figure 13, respectively. It is observed in Figure 12 that the high-temperature position is located at the corners on both sides of the rectangular radiator inlet. The low-temperature region corresponds to the bifurcation and inlet. With an increase in the AR, peak temperature initially increases and then decreases. In Figure 13, the temperature gradient decreases and the temperature uniformity improves with the increase in the AR. The region in which the temperature gradient changes sharply corresponds to the inlet and bifurcation of the channel.

**Figure 14:**
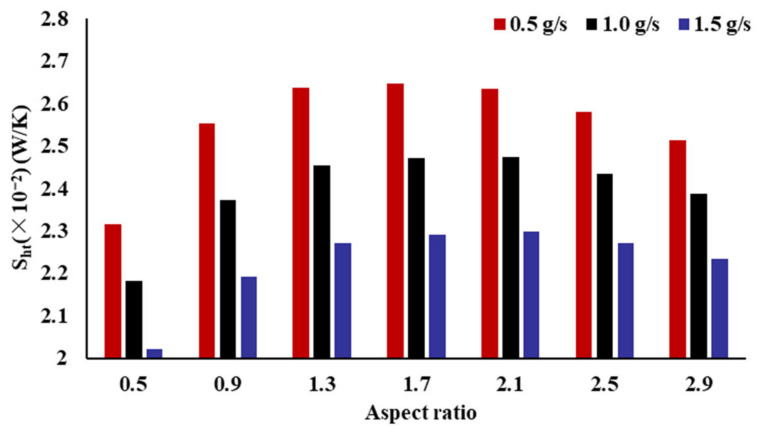
Effect of the inlet AR on entropy generation rate.

The irreversible loss degree is analyzed using the entropy generation rate ($S_{ht}$). Figure 14 shows the connection between the $S_{ht}$ and the AR of the radiator channel. It is observed that the AR has an evident impact on the $S_{ht}$. As the AR increases, the $S_{ht}$ first increases and then decreases. When the AR is 0.5, the fractal channel heat sink has the lowest entropy generation rate. The smaller the value of $S_{ht}$, the smaller the degree of irreversible loss. With different mass flow rates, the maximum differences in the entropy generation rates of the fractal-channel radiators with different ARs are 0.330 × 10^−2^, 0.293 × 10^−2^, and 0.277 × 10^−2^, with decreases of 12.48%, 11.82%, and 12.05%, respectively.

**Figure 15:**
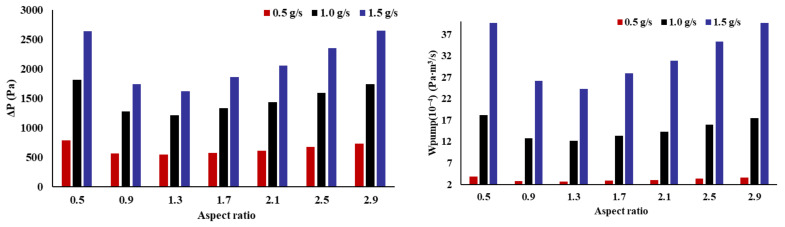
Effect of the inlet AR on pressure drop and pump power.

**Figure 16:**
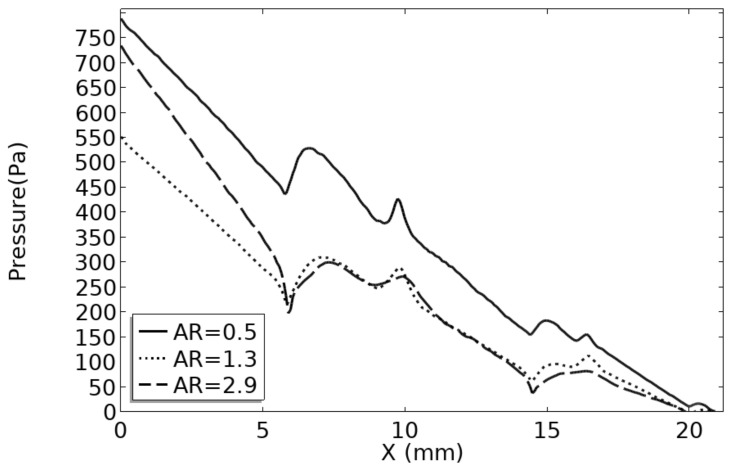
Fluid boundary pressure drop distribution.

It is observed in Figure 15 that the impact trend of the AR on the flow performance is different from that of the heat transfer performance. With an increase in the AR, the pressure drop (ΔP) and pump power initially decrease and then increase. The heat transfer performance is improved at the cost of greatly reduced flow performance. Since the power consumption is linearly related to the voltage drop, only the voltage drop is discussed below. The channel with an AR of 1.3 has the smallest pressure drop. In all cases, the minimum pressure drop is reduced by 30.38%, 33.17%, and 38.95%, respectively, compared with the maximum pressure drop. Figure 16 shows the pressure distribution of the channel with a partial AR. Pressure recovery occurs at the bifurcation. In the fractal channel, the one-level channel accounts for much of the pressure drop. The one-level channel with an AR of 2.9 has a large pressure drop and the two-level channel is smaller. Thus, the flow performance of the channel with an AR of 2.9 is poor in the one-level channel, but improves after entering the two-level channel.

**Figure 17:**
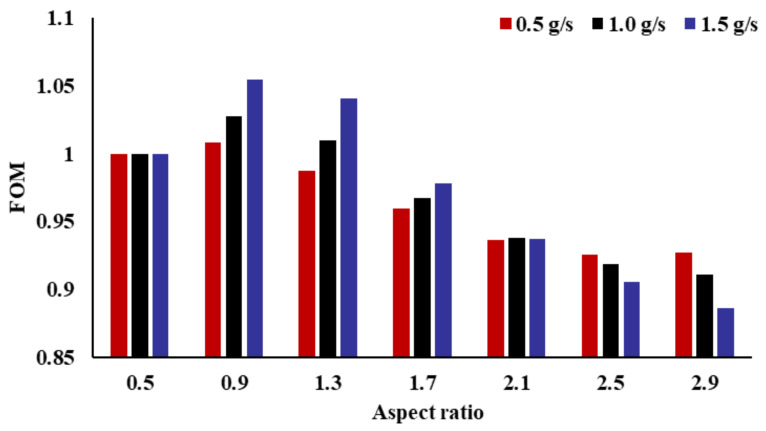
Effect of the inlet AR on FOM.

From previous research, it is found that AR has different effects on the thermal and flow performance of the radiator. To comprehensively evaluate the influence of the AR on the performance of the radiator, FOM is introduced. When the FOM is larger, it represents that the comprehensive performance of the radiator is better. Figure 17 shows the impact of the AR on the FOM of the radiator. With the increase in the AR, the FOM first increases and then decreases. When the channel AR is 0.9, the radiator has the largest FOM. When the channel AR is 2.9, the radiator has the smallest FOM. For the three mass flow rates, the maximum FOM increases by 8.78%, 12.88%, and 19.09%, respectively, compared to the minimum FOM. Thus, the fractal-channel radiator with an AR of 0.9 has the best comprehensive performance, and the radiator with an AR of 2.9 has the worst comprehensive performance.

### 4.2. Effect of All Levels Channel Aspect Ratio on Performance of Fractal Channel

The results in Section 4.1 show that the aspect ratio (AR) has an evident impact on the performance of the fractal channel. However, in Section 4.1, only the AR of the inlet can be controlled. It is necessary to control the ARs of all levels and study them. Therefore, the CARFC heat sink is discussed and compared with the optimal results of the IARFC heat sink.

**Figure 18:**
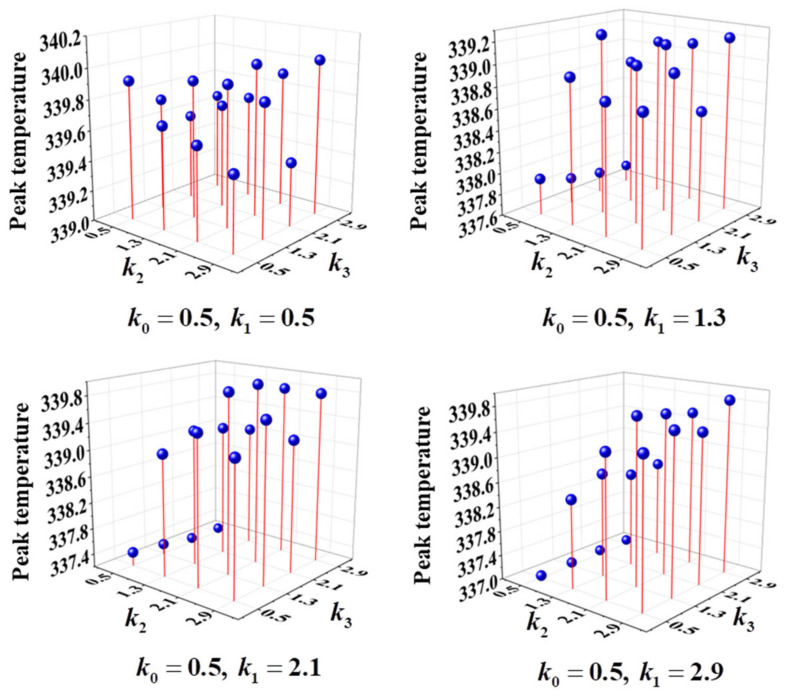

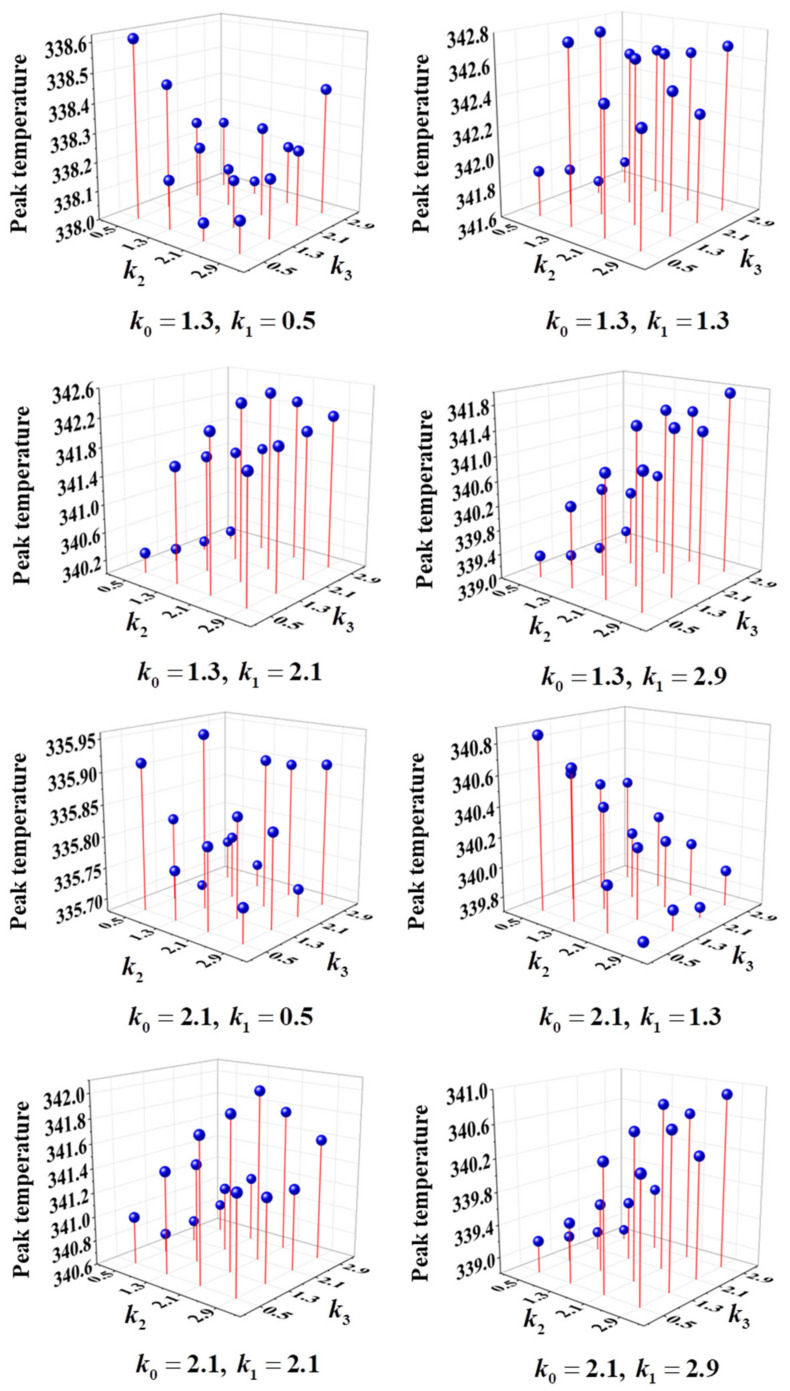

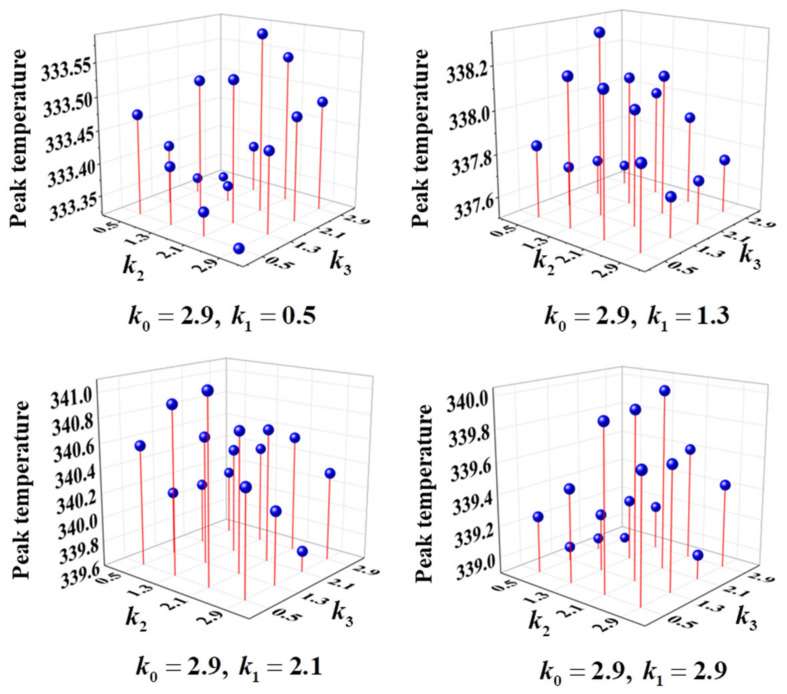
Peak temperature of CARFC heat sinks with different channel AR.

Figure 18 shows the peak temperatures ($T_{\max}$) of the CARFC heat sink with different ARs. It is observed that the AR has an important impact on the peak temperature of the heat sink. When the ARs are $k_{0}=1.3,k_{1}=1.3,k_{2}=1.3,k_{3}=1.3$, the peak temperature of the radiator is the largest (342.80 K). When the ARs are $k_{0}=2.9,k_{1}=0.5,k_{2}=2.9,k_{3}=0.5$, the peak temperature of the radiator is the smallest (333.32 K). The minimum value is 13.61% lower than the maximum value. Thus, the ability of the heat sink to suppress peak temperature can be significantly improved by adjusting the AR of each level channel.

**Figure 19:**
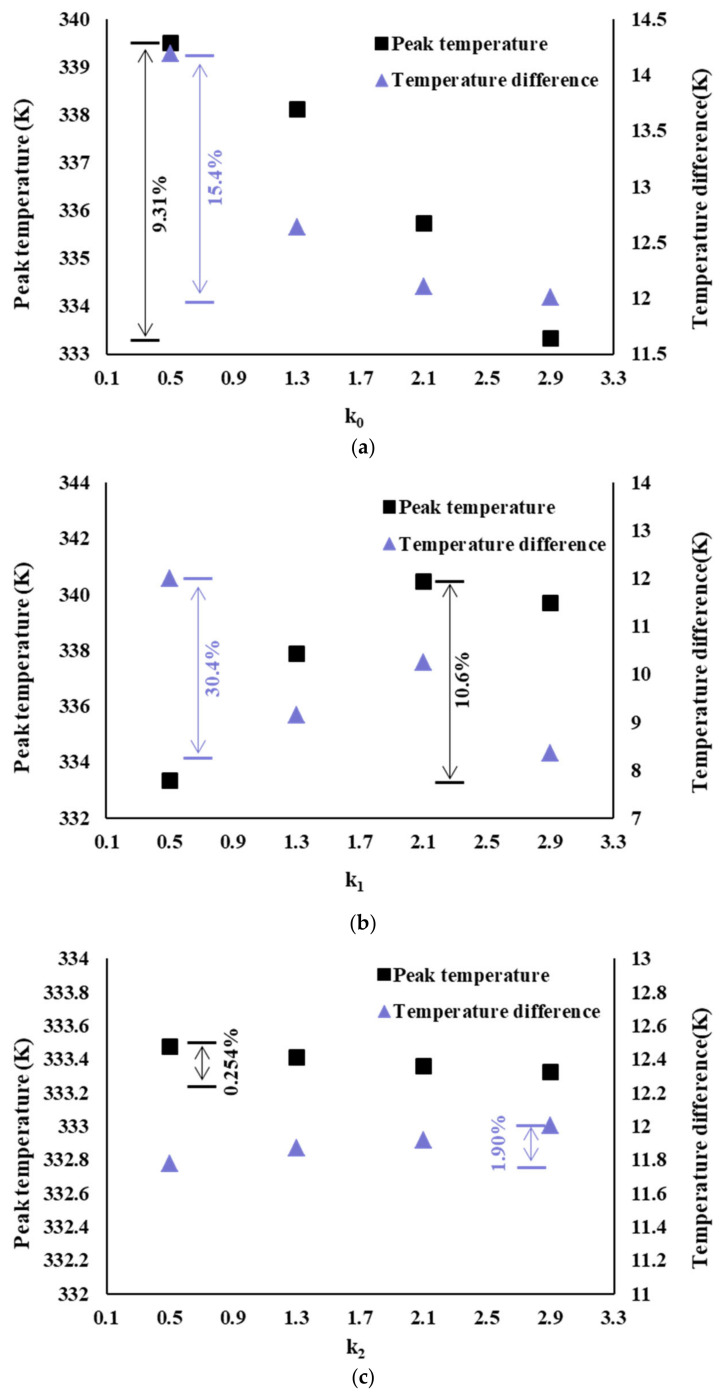

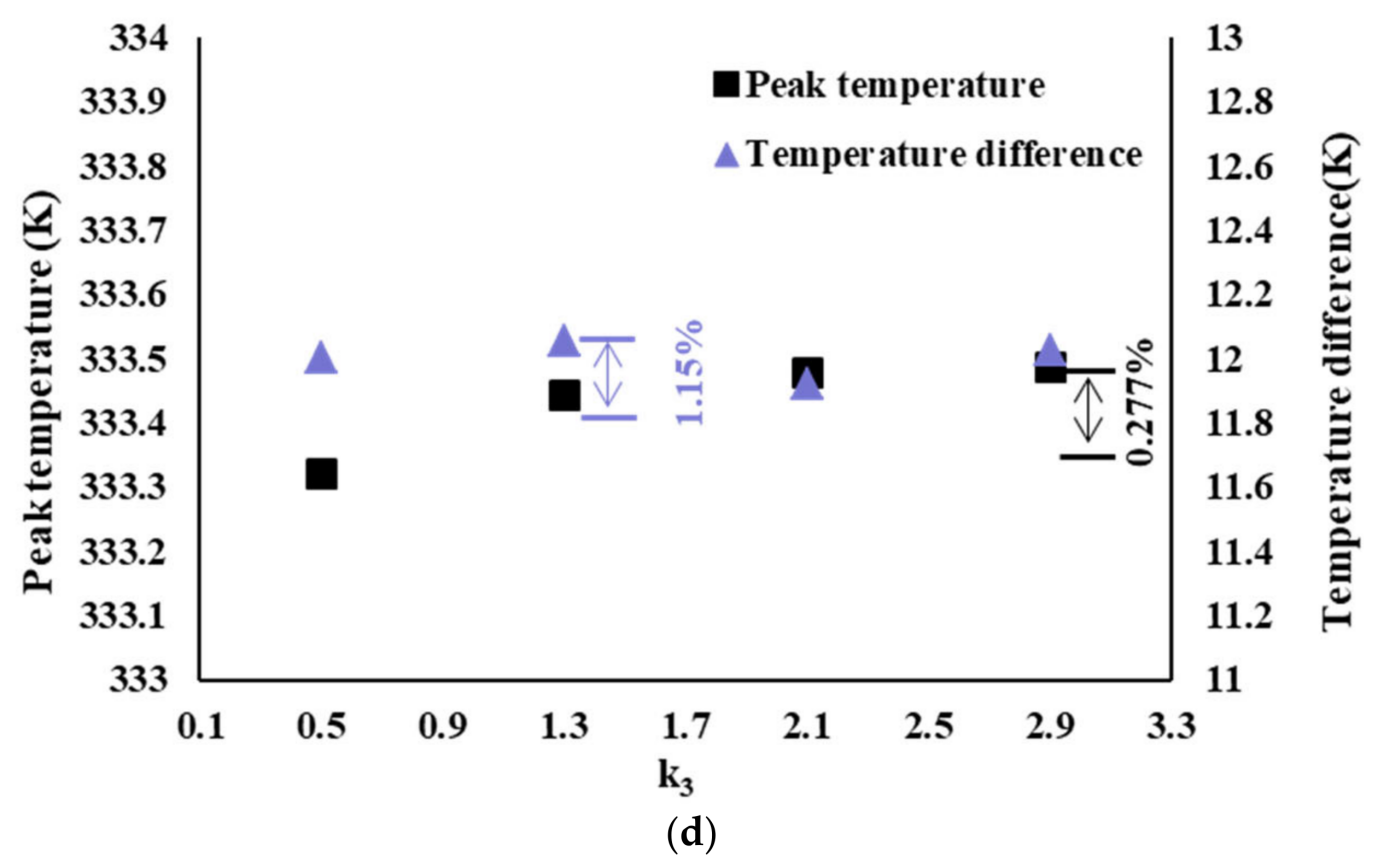
Influence of AR of each level channel on heat transfer performance heat sink: (**a**) The influence of $k_{0}$ on heat transfer performance. (**b**) The influence of $k_{1}$ on heat transfer performance. (**c**) The influence of $k_{2}$ on heat transfer performance. (**d**) The influence of $k_{3}$ on heat transfer performance.

To better understand the influence of the AR of each level channel on the heat exchange performance, the influence of the AR of the same channel on the heat exchange performance is compared on the premise that the AR of other channels remained unchanged. The results are shown in Figure 19. Figure 19a shows the influence of AR $k_{0}$ on the heat transfer performance of the fractal channel. The other ARs are $k_{1}=0.5$, $k_{2}=2.9$, and $k_{3}=0.5$. It can be seen that the peak temperature and temperature difference decrease with the increase in the AR. The maximum change rate of peak temperature is 9.31%. The maximum rate of change in temperature difference is 15.4%. Figure 19b shows the influence of AR $k_{1}$ on the heat transfer performance of the fractal channel. The other Ars are $k_{0}=2.9$, $k_{2}=2.9$, and $k_{3}=0.5$. With the increase in the AR, the peak temperature first increases and then decreases. When the AR is 2.9, the temperature difference of the heat sink is the smallest. The maximum change rate of peak temperature is 10.6%. The maximum rate of change in temperature difference is 30.4%. Figure 19c shows the influence of AR $k_{2}$ on the heat transfer performance of the fractal channel. The other ARs are $k_{0}=2.9$, $k_{1}=0.5$, and $k_{3}=0.5$. With the increase in the AR, the peak temperature decreases, but the temperature difference increases. The maximum change rate of peak temperature is 0.524%. The maximum rate of change in temperature difference is 1.90%. Figure 19d shows the influence of AR $k_{3}$ on the heat transfer performance of the fractal channel. The other Ars are $k_{0}=2.9$, $k_{1}=0.5$, and $k_{2}=2.9$. The peak temperature increases with the increase in aspect ratio. When the AR is 2.1, the temperature difference of the heat sink is the smallest. The maximum change rate of peak temperature is 0.277%. The maximum rate of change in temperature difference is 1.15%. In conclusion, among all levels channel ARs, $k_{0}$ and $k_{1}$ have a greater influence on the heat transfer performance of the fractal channel, while the other aspect ratios have little influence on the heat transfer performance.

**Figure 20:**
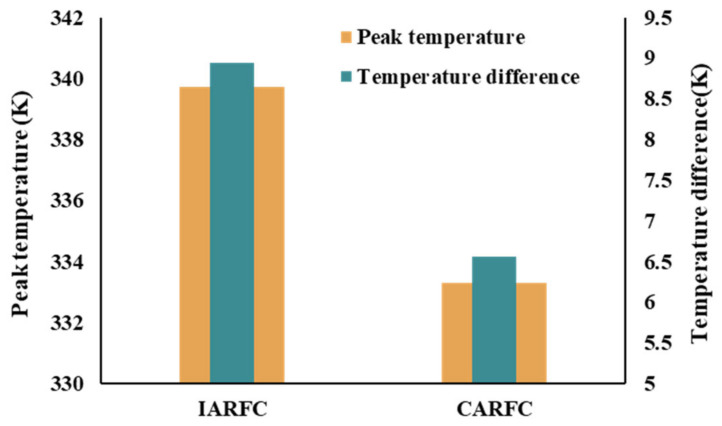
The Comparison results.

Figure 20 shows the comparison between IARFC and CARFC. It can be seen that CARFC greatly improves the heat transfer performance of the fractal channel by adjusting the AR of each level channel. In this part of the comparison, the AR selected for the comparison of the two performances is different, but the performance index corresponding to the selected AR is the best. When comparing the peak temperature of the two, the aspect ratio of IARFC is 0.5 and that of CARFC is $k_{0}=2.9,k_{1}=0.5,k_{2}=2.9,k_{3}=0.5$. When comparing the temperature difference between the two, the aspect ratio of IARFC is 2.9 and that of CARFC is $k_{0}=2.9,k_{1}=2.9,k_{2}=2.9,k_{3}=2.1$. Compared with the IARFC, the peak temperature of CARFC is reduced by 9.62% and the temperature difference is reduced by 26.57%.

## 5. Conclusions

To enhance the temperature uniformity and reduce the peak temperature, a controllable aspect ratio (AR) fractal channel (CARFC) heat sink is proposed to enhance thermal performance. First, a parameterized modeling method for the CARFC is constructed. Under a constant heat flow boundary, the effect of aspect ratio on the fractal channel performance is studied by numerical simulation. The conclusions obtained are as follows:

A parameterized modeling method for the CARFC is constructed. The AR of each level fractal channel can be controlled using this method. The control points and bifurcation points are constructed using the parameters, and a fractal channel is constructed. The channel size is determined by the AR of each level channel.

The inlet AR has an obvious influence on the performance of the fractal channel. With an increase in the AR, the Nusselt number and temperature difference decrease, and the entropy generation rate and thermal resistance show a non-monotonic trend of first increasing and then decreasing. The pressure drop shows the opposite trend, decreasing first and then increasing, and the comprehensive performance FOM of the fractal channel first increases and then decreases.

The ability of the heat sink to suppress peak temperature can be significantly improved by adjusting the AR of each level channel. It is found that when the AR is $k_{0}=2.9,k_{1}=0.5,k_{2}=2.9,k_{3}=0.5$, the peak temperature is the lowest, reduced by 13.61%. Among all levels channel ARs, $k_{0}$ and $k_{1}$ have a greater influence on the heat transfer performance of the fractal channel. Compared with only changing the aspect ratio of the inlet, the CARFC has better performance; the peak temperature and temperature difference are reduced by 9.62% and 26.57%, respectively.

## Figures and Tables

**Table 1:** Geometry information.

Substrate Length (L)	Substrate Width (W)	Channel Volume (V)	Levels Ratio	Pipe Diameter Ratio
20 mm	8 mm	17.5 mm^3^	2^−1/2^	2^−1/3^

**Table 2:** Materials of Y-shaped fractal channel heat sink.

Material	Specific Heat Capacity (J/(kg·K))	Thermal Conductivity (W/(m·K))	Density (kg/m^3^)
Aluminum	900	238	2700

**Table 3:** Boundary conditions.

Inlet Temperature	Heat Flux	Mass Flow Rate	Pressure of Outlet
293.15 K	500,000 W/m^2^	0.5, 1.0, 1.5 g/s	0 Pa

## Data Availability

Not applicable.

## Nomenclature

β	Levels ratio	$A_{h}$	Bottom area (m^2^)
n	Bifurcation levels	$R_{t}$	Total thermal resistances (K/W)
λ	Pipe diameter ratio	q	Heat flux (W/m^2^)
α	Bifurcation angle	ΔP	Pressure drop (Pa)
k	Aspect ratio (AR)	$D_{h}$	Hydraulic diameter (mm)
d	Channel width (mm)	$k_{l}$	Thermal conductivity of heat exchange fluid (W/(m·K))
h	Channel height (mm)	$T_{ave}$	Average temperature of heat sink (K)
L	Channel length (mm)	$T_{out}$	Outlet temperature (K)
W	Width of substrate (mm)	$T_{\max}$	Peak temperature of heat sink (K)
S	Area of inlet (mm^2^)	$T_{in}$	Inlet temperature (K)
V	Volume of channel (mm^3^)	$C_{p}$	Specific heat capacity (J/(kg·K))
m	Flow mass	ΔT	$T_{\max}-T_{min}$
Nu	Nusselt number	$V_{in}$	Volume flow rate
$S_{ht}$	Entropy generation rate	*FOM*	Figure of merit
h	Average coefficient of heat transfer	$W_{pump}$	Pumping power
$T_{\min}$	Minimum temperature of heat sink (K)		
